# Erythropoietin and Cancer: The Unintended Consequences of Anemia Correction

**DOI:** 10.3389/fimmu.2014.00563

**Published:** 2014-11-11

**Authors:** Nataša Debeljak, Peter Solár, Arthur J. Sytkowski

**Affiliations:** ^1^Faculty of Medicine, Institute of Biochemistry, University of Ljubljana, Ljubljana, Slovenia; ^2^Department of Cell and Molecular Biology, Institute of Biology and Ecology, Faculty of Sciences, Pavol Jozef Šafárik University, Košice, Slovakia; ^3^Oncology Therapeutic Area, Quintiles Transnational, Arlington, MA, USA

**Keywords:** erythropoietin, erythropoietin receptor, receptor partners, cancer, cell response, angiogenesis, clinical trials

## Abstract

Until 1990, erythropoietin (EPO) was considered to have a single biological purpose and action, the stimulation of red blood cell growth and differentiation. Slowly, scientific and medical opinion evolved, beginning with the discovery of an effect on endothelial cell growth *in vitro* and the identification of EPO receptors (EPORs) on neuronal cells. We now know that EPO is a pleiotropic growth factor that exhibits an anti-apoptotic action on numerous cells and tissues, including malignant ones. In this article, we present a short discussion of EPO, receptors involved in EPO signal transduction, and their action on non-hematopoietic cells. This is followed by a more detailed presentation of both pre-clinical and clinical data that demonstrate EPO’s action on cancer cells, as well as tumor angiogenesis and lymphangiogenesis. Clinical trials with reported adverse effects of chronic erythropoiesis-stimulating agents (ESAs) treatment as well as clinical studies exploring the prognostic significance of EPO and EPOR expression in cancer patients are reviewed. Finally, we address the use of EPO and other ESAs in cancer patients.

## Introduction

The presence of a circulating hemopoietic factor controlling the red blood cell (RBC) production was first suggested in 1906 (1). This humoral factor was experimentally confirmed almost half a decade later and the name *erythropoietin* was given (2). In 1977, the protein was isolated from human urine (3) enabling research toward cloning of the gene, its characterization, and *in vitro* expression (4, 5). Only 4 years later, the US Food and Drug Administration (FDA) approved the first commercially available recombinant human erythropoietin (rHuEPO), epoetin alfa, for the treatment of anemia associated with chronic kidney disease (CKD) (6). Later on, it was approved also for use in patients with other anemias including cancer patients undergoing chemotherapy (7). Thereafter, rHuEPO became a leading drug for treatment of anemia virtually abolishing the need for RBC transfusion in some types of anemia. As a result, since the 1990s, several new erythropoiesis-stimulating agents (ESA) have become available on the market or are under development [reviewed in Ref. (8)].

Erythropoietin (EPO) was first considered to have a single biological purpose and action – the stimulation of RBC growth and differentiation and, as such safe, for use in cancer patients. Slowly, scientific and medical opinion evolved, beginning with the discovery of an effect on endothelial cell growth *in vitro* (9) and the identification of EPO receptors (EPORs) on neuronal cells (10). We now know that EPO is a pleiotropic growth factor that exhibits an anti-apoptotic action on numerous cells and tissues, including malignant ones [reviewed in Ref. (11–13)]. In this article, we present a short discussion of EPO, its signaling, and its action on non-hematopoietic cells. This is followed by a more detailed presentation of both pre-clinical and clinical data that demonstrate EPO’s diverse actions on cancer cells as well as possible receptors involved in the response of cancer cells to EPO/ESA therapy. Finally, we review current recommendations for the use of rHuEPO and other ESAs as supportive therapy in cancer patients with anemia that often develops during the radio- or chemotherapy.

### Erythropoietin

The human *EPO* gene spans over 3 kb and contains five exons encoding a 193 amino acid protein (4, 5). It is a single copy gene located on chromosome 7 at position 7q22 (14, 15). A single splice variant of *EPO* gene is known (http://www.ncbi.nlm.nih.gov/gene/2056). Gene expression is regulated by oxygen levels through hypoxia. Transcription factors involved are stimulatory HIF-2, HNF-4alpha and inhibitory GATA-2, NF-kappaB [reviewed in Ref. (16, 17)].

During post-translation modification, the N-terminal 27 amino acid signal peptide is cleaved and R166 removed resulting in a 165 amino acid mature protein (18). Urinary protein containing 166 amino acids has also been characterized (19). The single-chain protein is heavily glycosylated with a molecular weight ranging from 30 to 39 kDa. Three N-linked (N24, N38, and N83) and one O-linked (S126) oligosaccharide side chains represent 35–40% of the EPO molecular mass. Protein structure is stabilized with two intra-chain disulfide bridges between C7–C161 and C29–C33 (19, 20). N glycosylation does not affect hormone function *in vitro* but is essential for *in vivo* biological activity like biosynthesis, structural stability, secretion, plasma half-life, and clearance (21–23).

In adult human beings, the hormone is produced mainly by the renal cortex (24, 25), while in the developing fetus, the liver is the principal source (26). EPO is secreted into the bloodstream, circulates to the bone marrow, and binds to EPOR situated on the cell surface of erythroid progenitors promoting their survival, proliferation, and differentiation (27). EPO is also produced by numerous non-hematopoietic cells and may act in endocrine, autocrine, and paracrine manner (28).

Commercially available rHuEPO has the same 165 amino acid sequence as naturally occurring hormone (29). However, the level of glycosylation in rHuEPO depends on the expression system used (30). Glycosylation pattern can be analyzed by isoelectric focusing enabling, thus distinguishing endogenous EPO (eEPO) from rHuEPO (31). Also, urinary and serum EPO have some minor heterogeneity in glycosylation levels (32).

### Erythropoietin signal transduction

**Figure 1:**
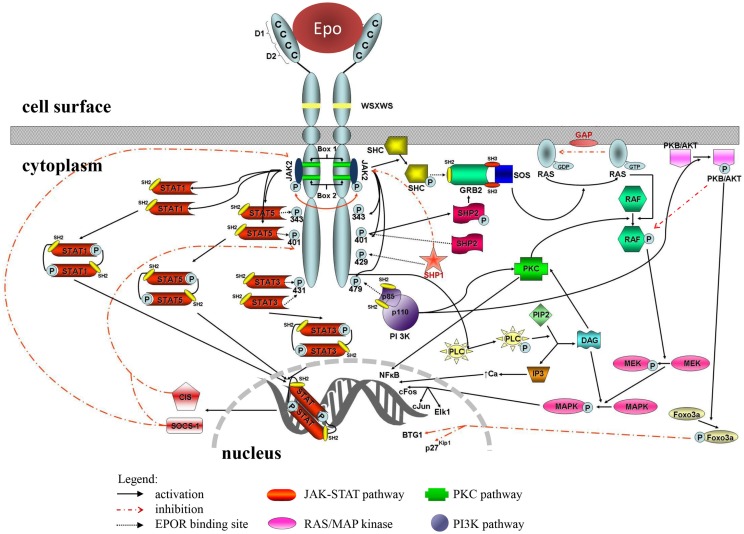
**Erythropoietin receptor and signaling pathways**. The structure of the receptor dimer is outlined; docking sites for several intracellular proteins are marked with P and linked with black-dotted arrow to individual pathway components. Positive interactions are presented with full black arrows, negative with dotted red.

In classical signal transduction in erythropoiesis, one EPO molecule binds to an EPOR homo-dimer leading to activation of the EPO-EPOR signaling cascade. EPO has two non-identical binding sites toward EPOR receptors, a high-affinity G151 (nano-molar) to the first, and a low-affinity R103 (micro-molar) interaction to the second receptor (33). Main signaling pathways activated by EPO are JAK2/STAT5 pathway, phosphatidylinositol 3-kinase (PI3K) pathway, RAS/MAP kinase pathway, and protein kinase C (PKC) pathway (34) (Figure 1). The JAK2/STAT5 and RAS/MAP kinase (RAS-RAF-MEK-ERK) pathways are associated with hormone mitogenic action, while the PI3K pathway (PI3K-AKT) is related with anti-apoptotic activities (27).

In non-hematopoietic tissue, some other receptor partners have been proposed, including the beta common receptor (βcR) (35) and the epinephrine B4 receptor (EPHB4) (36). The EPO molecule was indicated to bind to the hetero-dimmer EPOR-EPHB4 or hetero-trimmer EPOR-βcR-EPOR. Most probably, the high-affinity site is involved in binding to EPOR, while other receptor partners are bound with low affinity or an alternative site resulting in activation of different, tissue-protective part of EPO-EPOR signaling cascade.

#### Erythropoietin receptor

The human *EPOR* gene spans over 6 kb and contains 8 exons encoding a 508 amino acid protein (37). The gene is located on chromosome 19 at position 19p13.3-p13.2 (38, 39). Several *EPOR* splice variants are known such as non-coding RNA, functional transmembrane protein (EPOR-F), truncated protein (EPOR-T), and at least one soluble variant (EPOR-S) (40–42) (http://www.ncbi.nlm.nih.gov/gene/2057).

EPO receptor is a member of the type I cytokine receptor superfamily (43). During post-translation modification, the N-terminal 24-amino acid signal peptide is cleaved and the protein is modified by glycosylation, phosphorylation, and ubiquitination to a mature 66–105 kDa protein (38, 44, 45). Mature human receptor consists of extracellular, single transmembrane, and cytoplasmic regions (46) (Figure 1).

The majority of the EPOR is located on the cell surface of erythroid progenitors, erythroid burst-forming units (BFU-E), and erythroid colony-forming units (CFU-E) in the bone marrow. However, the EPOR is expressed by various other tissues such as brain, heart, liver, and others where it is involved in the tissue protection. As EPOR is present in various cancer cells, the use of rHuEPO in cancer patients may be problematic due to potential activation of EPO-EPOR signaling pathways resulting in tumor protection (anti-apoptotic action) or proliferation (mitogenic action).

#### Beta common receptor (βcR)

The human *colony-stimulating factor 2 receptor, beta (CSF2RB)* gene contains 14 exons and is located on chromosome 22 at position 22q13.1 (http://www.ncbi.nlm.nih.gov/gene/1439). *CSF2RB* gene encodes β-common receptor (βcR), a common beta chain subunit of the high-affinity receptor for interleukin 3 (IL3), interleukin 5 (IL5), and CSF2 (granulocyte-macrophage colony-stimulating factor).

**Figure 2:**
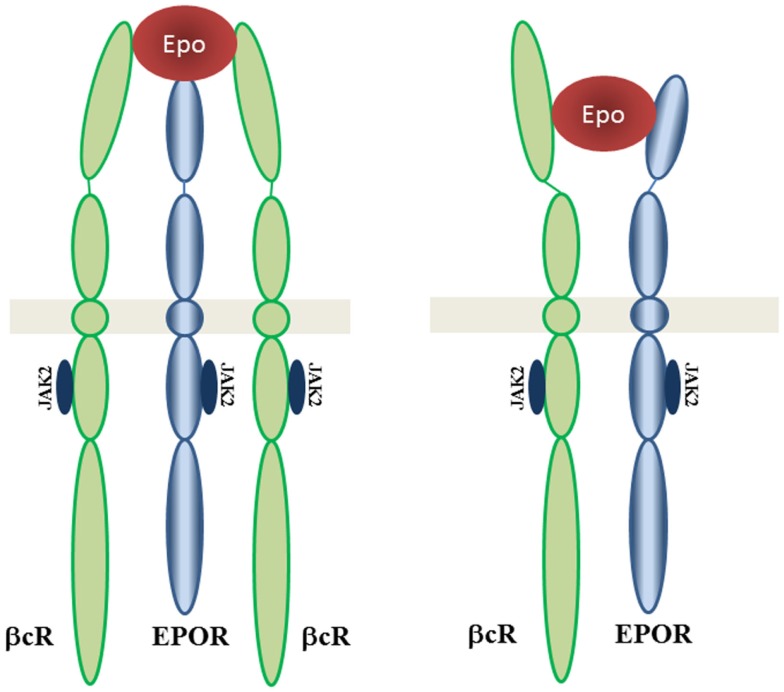
**EPOR-βcR receptor complex**. Proposed structures of tissue-protective complexes are presented: heterotrimer including βcR homodimer with EPOR and heterodimer βcR with EPOR. Adapted from Brines and Cerami (47).

The interaction of EPOR with the βcR was discovered and tissue protection may be signaling through a heteroreceptor complex involving EPOR and βcR (35). This signaling network is still not understood but may involve signal pathways different from those triggered by EPOR-EPOR (47–50). The tissue-protective effects of EPO could be mediated by an EPOR heteroreceptor dimer EPOR-βcR or trimmer EPOR-βcR-EPOR (Figure 2).

#### Ephrin type-B receptor 4 (EPHB4)

The human *Ephrin receptor B4 (EPHB4)* gene contains 17 exons and is located on chromosome 7 at position 7q22 (http://www.ncbi.nlm.nih.gov/gene/2050). Ephrin receptors were named Eph after the EPO-producing hepatocellular carcinoma cell line from which its cDNA was isolated. They form the largest family of receptor tyrosine kinase (RTK) family. About 16 ephrin receptor genes (EphA1-10, EphB1-6) have been identified in the vertebrate genome (51), 14 of which are present in human beings. Ephrin receptors and their ligands, the ephrins, mediate numerus developmental processes. The protein encoded by *EPHB4* gene binds to ephrin-B2 ligand and plays an essential role in vascular development. EPHB4 was indicated also as survival factor in several cancers (52, 53).

**Figure 3:**
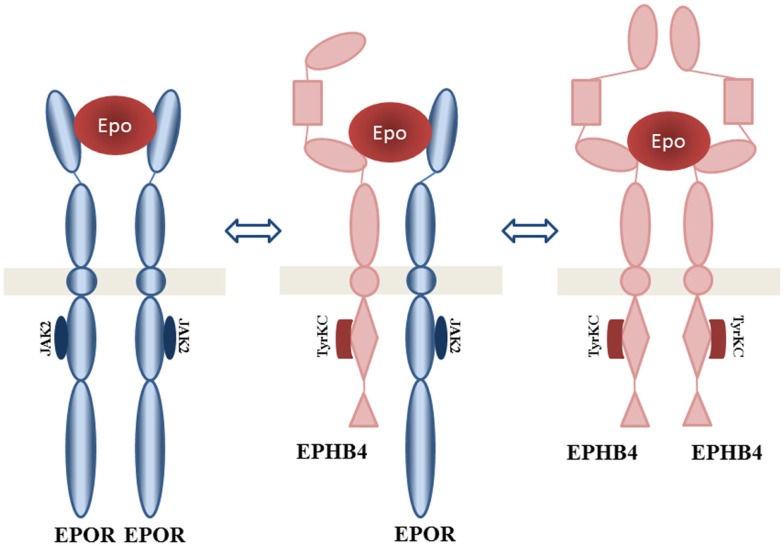
**EPOR-tissue-protective erythropoietin receptor (nepor receptor complex)**. Proposed structure of EPO interacting complexes are presented: homodimer EPHB4, homodimer EPOR, and heterodimer EPHB4 with EPOR. Adapted from Jackson (36).

European patent application [EP 2 492 355 A1 (36)] discloses a molecular composition(s) of a novel tissue-protective EPO-binding receptor protein complex, termed NEPOR. NEPOR represents a novel EPOR derived from a unique combination of EPOR heteroreceptor dimer EPOR-EPHB4, homoreceptor EPOR-EPOR, and EPHB4-EPHB4, and other components derived from ephrin biology (Figure 3).

#### Other partners

Interaction of EPOR with several receptors has been indicated. Some interactions were suggested only on the level of receptor expression correlation (54, 55), others on adverse tumor response to receptor-specific therapy (56, 57). The exact mechanisms of these correlations is not known yet but are being intensively studied.

Increased EPOR expression was related to a reduced response to tamoxifen treatment, an estrogen receptor (ER) specific inhibitor (56). Furthermore, EPOR receptor was shown to enhance ER activity and promote cell proliferation (58). Several correlations between EPOR, ER, and other steroid receptors expression have been described (54, 55, 59, 60).

The antagonistic effect of EPOR and human epidermal growth factor receptor-2 (HER2) co-expression on transtuzumab therapy, a HER2 inhibiting antibody has also been observed (57). The JAK2-mediated activation of SRC and inactivation of PTEN have been proposed as underlying mechanisms.

## Action of EPO on Non-Hematopoietic Cells

EPO and its receptor have been identified in several non-hematopoietic cells and tissue types like central nervous system, heart, kidney, gastrointestinal system, reproductive tract, endothelium, and others [reviewed in Ref. (11, 12, 61)]. In these tissues, EPO was shown to be tissue protective in an anti-apoptotic and/or mitogenic manner. Several ongoing pre-clinical and clinical trials are exploring the potential use of rHuEPO and other ESAs, such as tissue-protective agents in the brain, heart, and wound healing [reviewed in Ref. (62, 63)].

Furthermore, EPORs have been found also on several types of tumors and malignant cells [reviewed in Ref. (64–67)] questioning the use of recombinant EPO in cancer patients (68). We will now focus on a review of various effects of EPO and its receptor on cancer cells, being an update of EPO research reviewed by Szenajch et al. (13).

### EPO and cancer cells response

The presence of functional EPOR was demonstrated in cancer stem cells (69–71). Different types of tumors as well as cell lines have been found to express *EPOR* mRNA transcripts, which might be translated into full-length EPOR as well as soluble or other truncated forms (40). In this regard, Um et al. (72) measured internalized ^125^I-EPO and found that just 50 high-affinity cell surface EPO binding sites were sufficient for EPO-mediated activation of intracellular signal transduction in SH-SY5Y and PC-12 cancer cells. EPOR expression has been demonstrated by flow cytometry using a specific EPOR antibody in a panel of 29 tumor cell lines, including 18 adherent cell lines (73).

Despite the fact that many tumor cells were confirmed to possess the EPOR, there is still some debate on stimulatory effects of EPO on these cells. On the one hand, there are papers pointing to the proliferative response of cancer cells after rHuEPO treatment (55, 74–82); on the other hand, some tumor cells, in spite of evidence of EPOR functionality (67, 81), did not exhibit a growth response (67, 83–86). Our previous studies revealed a weak surface EPOR signal in A2780 cells with most of EPOR found in the cytoplasm, more abundantly as an intracellular membrane-associated protein than a soluble one. Silencing EPOR expression resulted in reduced A2780 proliferation as well as a reduction in EPO-induced phosphorylation of ERK1/2 (81). Unlike hematopoietic cells, where EPO-EPOR signaling is associated with increased cell proliferation and/or survival, in tumor cells, the EPO-EPOR axis does not always lead to increased proliferation but might increase the resistance of cancer cells to different therapies. For more information about the role of EPO and its receptor in growth, survival, and therapeutic response of human cancer cells, see the critical review of Szenajch et al. (13), where all information based on published EPO papers through 2010 is well summarized.

Recently, the presence of EPOR signaling and EPO-induced cellular proliferation was confirmed in renal cancer cells (80, 82), head and neck squamous cell carcinomas (77), and cervical cancer cell lines (78), as well as glioma cells (87).

Erythropoietin-induced proliferation of cancer cells was associated with the activation of JAK2, JAK3, STAT3, and STAT5 but not JAK1 or STAT1 (78), AKT phosphorylation (77), ERK phosphorylation (87) with hTERT gene transcription by JAK2/STAT5/c-MYC, and hTERT protein phosphorylation by PI3K/AKT (88). Furthermore, the EPO-EPOR pathway stimulated the expression of cyclin D1 and inhibiting the expression of p21cip1 and p27kip1 through the phosphorylation of JAK2 and ERK1/2, led to a more rapid progression through renal cancer cell cycle (82). Interestingly, EPO or stem cell factor (SCF) alone produced a modest number of cervical cancer cell colonies, whereas the combination EPO/SCF induced a significantly more. Similarly, co-stimulation with EPO/SCF induced a significantly higher number of migrating cervical cancer cells than either cytokine alone. Concurrently, EPO induced a modest, transient activation of ERK1/2, whereas SCF and EPO/SCF prompted a strong, sustained phosphorylation of ERK1/2 (89).

Erythropoietin was also involved in cell growth, invasion, survival, and sensitivity to the multikinase inhibitor sunitinib and cisplatin in renal cancer cells (80) and in head and neck squamous cell carcinoma (77), respectively. *In vitro*, EPO had a protective effect on radiation-treated MDA-MB-435 cells; however, EPO treatment alone or combined with chemotherapy or hypoxia did not influence cell survival. *In vivo*, EPO increased lung metastases in immunocompromised mice injected with MDA-MB-231 or MDA-MB-435 cells and treated with chemotherapy relative to mice treated with chemotherapy alone (90).

Very recently, Trošt et al. (55) confirmed the results of Arcasoy et al. (91) with positive response of breast cancer cells to EPO. Moreover, they demonstrated time- and concentration-dependent manner of EPO-induced MCF-7 proliferation and EGR1, FOS, and EPOR as transcription targets of the EPO-EPOR signaling loop (55). In this regard, Inbar et al. (92) discovered that EPO-driven EGR1 and c-FOS gene expression, as well as histone H4 acetylation in breast cancer cells were mediated via polyADP-ribosylation. EPO-induced breast cancer cell migration was blocked by the PARP inhibitor Veliparib (ABT-888), suggesting an essential role for polyADP-ribosylation in this process and suggesting a new cancer-associated anemia treatment modality with combined administration of EPO and PARP inhibitors (92).

Recent research revealed that EPO/EPOR contributes to the mechanism of trastuzumab resistance in breast cancer cell line SKBR3, and EPOR downregulation can reverse the resistance to trastuzumab. EPOR expression may be involved in tumor progression and proliferation in HER2-positive breast cancer (93). Indeed, EPO antagonized trastuzumab-induced therapeutic effects through JAK2-mediated activation of SRC and inactivation of PTEN protein, so combined therapy of HER2-positive cancer cells with EPO and trastuzumab reduced the response of these cells to trastuzumab both *in vitro* and *in vivo*. Furthermore, concurrent administration of EPO and trastuzumab correlated with shorter progression-free and overall survival in patients with HER2-positive metastatic breast cancer (57). The mechanism of EPO-EPOR and HER2 co-regulation in breast cancer was confirmed by miR-125b, which is downregulated in metastatic breast cancers and a significant positive correlation between EPOR and HER2 levels that are both targets of miR-125b was demonstrated (94).

Because of adverse tumor response and/or poorer survival in ESA-treated cancer patients, studies of EPO effects on cancer stem cells was initiated. Cao et al. (69) found that glioma stem cells (GSC) express higher levels of EPOR than matched non-stem glioma cells. They targeted EPOR expression in GSG with shRNA and reduced growth, survival, and neurosphere formation capacity, so confirmed the role for EPOR in GSC maintenance. A small molecule inhibitor of STAT3 led to reduced GSG growth and survival. EPO-EPOR signaling was also critical for survival *in vivo*, as targeting EPOR expression decreased GSC tumorigenic potential (69). Furthermore, Todaro et al. (71) showed that breast cancer stem-like cells (BCSC) isolated from patient tumors express the EPOR and respond to EPO treatment with increased proliferation and self-renewal. Importantly, EPO stimulation increased BCSC survival and resistance to chemotherapeutic agents, probably by EPO-activated AKT and ERK pathways and promoted metastatic progression of tumor xenografts in the presence and in the absence of chemotherapy treatment. These results suggest that EPO acts directly on BCSC by activating specific survival pathways, resulting in BCSC protection from chemotherapy and enhanced tumor progression (71). Moreover, EPO/EPOR promoted tumorigenesis in genetically engineered mouse models of breast cancer by activating JAK/STAT signaling in breast tumor-initiating cells (TICs) and promoted its self-renewal. *EPO* gene expression correlated with shortened relapse-free survival and pharmacologic JAK2 inhibition revealed a synergistic effect with chemotherapy in tumor growth inhibition *in vivo* (70).

### EPO and tumor angiogenesis and lymphangiogenesis

In 1990, Anagnostou et al. (9) found that EPO enhances the proliferation and migration of human umbilical vein endothelial cells and bovine adrenal capillary endothelial cells (95, 96) and demonstrated the presence of *EPOR* mRNA in human umbilical vein endothelial cells, as well as strong positive EPOR protein staining of the vascular endothelium *in vivo* (97). The presence of EPOR was also shown by Yamaji et al. (98), who suggested that brain capillary endothelial cells express not only an authentic form of EPOR (EPOR-F) but also a soluble one (EPOR-S) and that EPO acts directly on brain capillary endothelial cells as a competence factor. EPO signaling as a mitogen of endothelial cells was conducted via tyrosine phosphorylation of proteins including phosphorylation of transcription factor STAT-5, which is similar to that occurring in erythroid cells (99). Moreover, experiments performed in cultured vascular cells demonstrated that EPO robustly induced phosphorylation of STAT-5 in HUVEC cells, but only very weakly in smooth muscle cells (100). Results of our group revealed that conditioned media of EPO treated A2780 cells under hypoxic conditions induced significant STAT-5 phosphorylation, as well as proliferation of HUVEC cells. A new finding is the fact that pro-stimulatory effect of hypoxic A2780 media was partly mediated by EPO. Furthermore, EPO increased secretion of IL-4, IL-5, IL-6, IL-8, IL-10, IL-12, IL-13, GM-CSF, and IFN γ by A2780 cells in hypoxic conditions (101).

An *in vivo* angiogenic potential of EPO was originally demonstrated by Yasuda et al. (102) who found that injection of EPO into the ovariectomized mouse uterine cavity promoted blood vessel formation of the endometrium. Similarly, Ribatti et al. (103) demonstrated that EPO induced a potent *in vivo* angiogenic response of the chick embryo chorioallantoic membrane. Furthermore, the role of EPO in physiological angiogenesis was described during wound healing and in the developing of mouse embryo (104, 105).

The study of Yasuda et al. (106) revealed that normal human cervix and endometrium, as well as ovary malignant tumors of female reproductive organs produce EPO and EPOR, and that the tumor cells themselves and capillary endothelial cells are sites responsive to the EPO signal. Yasuda et al. (107) proposed the presence of a paracrine or autocrine EPO-EPOR loop and its contribution to tumorigenesis in female reproductive organs based on the mitogenic action of EPO as well as on the finding that injection of soluble EPOR (EPOR-S) or EPO-monoclonal antibody into blocks of tumor specimens was followed by apoptosis of tumor cells and endothelial cells. Although some studies have not confirmed a direct stimulatory effect of EPO on tumor cells, there is ample evidence of this effect on endothelial cell proliferation and/or angiogenesis of tumors. In this regard, EPO xinduced angiogenesis in chemically induced murine hepatic tumors (108) and accelerated the growth of EPOR negative Lewis lung carcinoma cells by promoting tumor angiogenesis *in vivo* (109).

Interestingly, an EPO analog stimulated neovascularization in colorectal liver metastases of hepatectomized and non-hepatectomized mice (110). Moreover, Nico et al. (111) demonstrated that EPO secreted by glioma tumor cells affected glioma vascular endothelial cells and promoted angiogenesis in a paracrine manner. Specificity of the EPO effect was shown through an anti-EPO antibody, which was able to significantly inhibit the angiogenesis response. Despite the absence of melanoma growth stimulation *in vivo*, EPO increased vascular size in the xenografts. Indeed, EPO-induced angiogenesis in Matrigel plug assays, and neutralization of EPO secreted by melanoma cells resulted in decreased angiogenesis, which supports the role of EPO/EPOR in melanoma progression via angiogenesis stimulation (112). Even more interestingly, EPO accelerated the tumor growth of MMQ pituitary adenoma xenografts lacking EPOR via enhancement of angiogenesis *in vivo*, without a direct EPO effect on MMQ cells *in vitro*. EPO administration increased phosphorylation of JAK2, STAT3, and VEGF expression in HUVEC cells *in vitro* and in MMQ cell xenografts *in vivo* (113). The authors suggest that EPO administration may promote the growth of pituitary adenomas by enhancing angiogenesis through EPO-JAK2-STAT3-VEGF signaling pathway and should be used with caution in anemia patients bearing pituitary adenoma due to its potential deleterious effects (113). On the contrary, very recently Pascual et al. (114) found that preoperative administration of EPO stimulates tumor recurrence in an animal model of colon cancer, but no evidence of increased angiogenesis or enhanced-cell proliferation as possible mechanisms of EPO-induced recurrence was seen. Importantly, EPO/EPOR levels correlated well with angiogenesis and progression of patients with hepatocellular carcinoma, neuroblastoma, squamous cell carcinoma of the tongue, melanoma, and gastric adenocarcinoma (115–120).

The lymph node as a new target of EPO was presented by Lee et al. (121) They showed that EPO can stimulate both lymph node lymphangiogenesis and nodal metastasis by increased migration, capillary-like tube formation, and dose- and time-dependent proliferation of human lymphatic endothelial cells in tumor-bearing animals. Intraperitoneal administration of EPO induced AKT and ERK1/2 signalization followed by peritoneal lymphangiogenesis stimulation. Furthermore, systemic treatment of EPO increased infiltration of CD11b(+) macrophages in tumor-draining lymph nodes and increased VEGF-C expression in lymph node-derived CD11b(+) macrophages, as well as in bone marrow-derived macrophages in a dose- and time-dependent manner (121).

According to McKinney and Arcasoy (122), there are two potential mechanisms by which rHuEPO therapy may promote tumor progression and reduce survival in some cancer patients: (1) rHuEPO therapy may exert *local* effects in tumors, acting directly on tumor cells or other cell types in the tumor microenvironment, such as the vascular endothelium and tumor-associated macrophages or (2) rHuEPO may cause *systemic* effects that indirectly alter tumor biology in an unfavorable manner or directly give rise to specific systemic toxicities that impair survival. In this regard, elevated hemoglobin, increased viscosity, platelet activation, endothelial progenitor mobilization, immunomodulatory effects, and others could play significant roles. We add a third potential mechanism and this is *direct* effect of EPO on cancer stem and/or TICs, which could explain enhanced tumor progression and poor survival of some cancer patients treated with EPO.

### EPO (ESA) and clinical studies

Several clinical trials have addressed the effects of one of the EPO/ESA treatments on disease control in cancer patients on co-temporal anti-cancer therapy, such as chemotherapy, radiotherapy, neoadjuvant therapy, and surgery. Such EPO/ESA treatments are long termed and rarely single dosed. The review of clinical trials performed between 2009 and 2014 is presented in Table 1, being an update of review by Szenajch et al. (13). Some of the studies indicate a negative outcome (marked in gray). Most of the reported negative effects are due to increased thrombotic events, a complication driven by an increased number of RBC (123–134). However, some of the studies also indicate a reduced recurrence free survival and overall survival (128, 129, 135–137). Negative effects on overall survival were already shown previously by several studies, reviewed by Szenajch et al. (13).

**Table 1:** **Clinical trials with reported effects of ESA in cancer patients performed between 2009 and 2014**.

Study (reference)	Type	No. of patients (cancer type)	Therapy	Support therapy	Hb control	Disease control	Observations
Mäenpää et al. (138)		*N* = 114 (various)		EB			↑HRQoL
Thomaidis et al. (139)	Ph2	*N* = 118 (esophagogastric)	Cx	EB	↑Hb (ESA)	↑OS, RFS (ESA)	
AGO-ETC Moebus et al. (123)		*N* = 1.284 (breast)	Cx	EA	↑Hb, ↓TF (ESA)	↔OS, RFS	↑TE (ESA)
GINECO Weber et al. (140)	Ph2	*N* = 100 (ovarian)	Cx	EA			↓NT
Ohashi et al. (141)	Meta	*N* = 511 (CIA, various)	Cx	DP, EB	↓TF (ESA)	↔OS, RFS	↔TE
Michallet et al. (142)		*N* = 107 (leukemia)	Cx, T	ESA	↑Hb, ↓TF (ESA)	↔OS, RFS	↑HRQoL (ESA), ↔TE
Tonia et al. (124)	Meta	*N* = 20.102, 91 trials		ESA	↓TF (ESA)		↑TE, HRQoL (ESA)
Stehman et al. (143)		*N* = 1.864 (ovarian)	Cx	G-CSF, ESA		↔OS	multivariate analysis
CHOICE Aerts et al. (144); Van Belle et al. (145, 146)		*N* = 1.887 (various)	Cx	DP	↑Hb (ESA)		Five severe drug reactions
Kerkhofs et al. (147)		*N* = 113	Cx	EA	↑Hb (ESA)		NR
Canon et al. (125)	Ph3	*N* = 705	Cx	DP	↑Hb (ESA)		↑TE (ESA)
Bustos et al. (126)		*N* = 685 (CIA, various)	Cx	DP	↑Hb (ESA)		↑TE (ESA)
Cabanillas et al. (148)		N = 109 (leukemia, lymphoma)	Cx	EA	↓TF (ESA)	↔CR	↔HRQoL
Cantrell et al. (149)		*N* = 343 (CIA, ovarian)	Cx	ESA		↔OS, PFS	
Fujisaka et al. (127)	Ph3	*N* = 186 (CIA, various)	Cx	EB	↑Hb (ESA)	↔OS	↑HRQoL, ↑TE (ESA)
NOGGO-AGO Blohmer et al. (150)	Ph3	*N* = 257 (cervical)	Cx, Rx	EA	↑Hb (ESA)	↑RFS (ESA), ↔OS	↔TE
PREPARE Untch et al. (128, 129)	Ph3	*N* = 733 (breast)	Cx, Na	DP	↑Hb (ESA)	↓RFS (ESA)	↑TE (ESA)
Nagel et al. (151)	Ph2	*N* = 74 (SCLC)	Cx	DP	↓TF (ESA)	↔PFS	
RETRA Eisterer et al. (152)		*N* = 309 (various)	Cx	DP	↓TF (ESA)		
Djavan et al. (153)		*N* = 1.567 (prostate)	S	ESA		↔RFS	
Villegas et al. (130)	Ph2	*N* = 44 (MDS)		DP			↑TE (ESA)
Rørth et al. (154)		N = 16 (solid)	Cx	DP	↑Hb (ESA)		↑HRQoL (ESA)
Tjulandin et al. (155)		*N* = 186 (CIA, various)	Cx	ET	↑Hb, ↓TF (ESA)		↔AE
Esquerdo et al. (156)		*N* = 100 (CIA, solid)	Cx	DP	↑Hb, ↓TF (ESA)		
Chavez-MacGregor et al. (131)	Retro	*N* = 2.266 (breast)	Cx	ESA			↑TE (ESA)
Gómez et al. (157)		(gastrointestinal)	Cx	EB	↑Hb (ESA)	NR	
Gomez-Alamillo et al. (158)		*N* = 22 (solid)	Rx, Na	EB	↑Hb (ESA)		
Schwartzberg et al. (159)	Ph2	*N* = 752 (CIA, various)	Cx	DP	↑Hb (ESA)		↔AE
Ray-Coquard et al. (160)		*N* = 2.912 (solid)	Cx	DP	↑Hb (ESA)		
Pronzato et al. (161)		*N* = 223 (breast)	Cx	EA	↑Hb (ESA)	↔OS, TR, AE	↑PRO, HRQoL (ESA)
Vargas et al. (162)	Ph4	*N* = 157 (CIA, various)	Cx, Rx	EA	↑Hb, ↓TF (ESA)	↑AE (ESA)	
Auerbach et al. (163)	Ph2	*N* = 242 (solid)	Cx,	DP, Fe	↑Hb (Fe)	↔AE	
Maccio et al. (164)		*N* = 148 (various)	Cx	EB, Fe	↔Hb (Fe)		
Ichinose et al. (165)	Ph2	*N* = 132 (lung, ovarian)	Cx	DP	↑Hb (ESA)		↑HRQoL (ESA)
GHSG HD15EPO Engert et al. (166)		*N* = 1.379 (HL)	Cx	EA	↓TF (ESA)	↔OS, RFS, PFS,	↔TE
Gascon et al. (135)	Ph2	*N* = 153 (NSCLC)	Cx	CERA, DP	↔Hb	↓OS (ESA)	Terminated early
Muravyov et al. (167)		*N* = 40 (solid)	Cx	EB	↑Hb (ESA)		
Stull et al. (168)		*N* = 1.494 (various)	Cx	DP	↑Hb (ESA)		↑HRQoL (ESA)
Tjulandin et al. (169)		*N* = 223 (CIA, various)	Cx	ET, EA	↑Hb (ESA)		
Roddy et al. (132)		*N* = 79 (colorectal)	Cx	ESA			↑TE (ESA)
Hoskin et al. (170)		*N* = 301 (head and neck)	Rx	EA	↑Hb (ESA)	↔OS, RFS	↔AE
Vansteenkiste et al. (171)	Ph3	*N* = 705 (CIA, solid)	Cx	DP	↑Hb, ↓TF (ESA)		
Grobmyer et al. (172)		*N* = 40 (abdominal)	S	EA	↔TF		
BRAVE Aapro et al. (133)		*N* = 463 (breast)	Cx	EB, At			↑TE (ESA), ↓TE (ESA, At)
Aapro et al. (134)	Meta	*N* = 2.297 (breast)	Cx	EB		↔OS	↑TE (ESA),
Hernandez et al. (173)	Ph3	*N* = 386 (CIA, solid)	Cx	DP	↓TF (ESA)		↔HRQoL, TE,
Greenberg et al. (174)	Ph3	*N* = 110 (MDS)	Cx	ESA, GCSF		↔OS	↑HRQoL (ESA)
Repetto and CIPOMO Investigators (175)		*N* = 1.175 (various)	Cx	ESA, GCSF			
Bohlius et al. (136)	Meta	*N* = 13.933 (various)		ESA		↓OS (over ↑Hb)	
Lambin et al. (137)		*N* = 1.397 (head and neck)	Cx, Rx	ESA		↓OS (over ↑Hb)	
Tzekova et al. (176)	Ph3	*N* = 216 (solid)	Cx	EZ	↑Hb (ESA)		↓TE (EZ), ↑HRQoL (ESA)

**Study type*: Meta, meta-analysis; Ph2-4, clinical study phase 2–4; Retro, retro-analysis. *Disease/cancer type*: CIA, chemotherapy-induced anemia; HL, Hodgkin’s lymphoma; MDS, myelodysplastic syndrome; NSCLC, non-small-cell lung cancer; SCLC, small-cell lung cancer. *Therapy*: At, antithrombotic; CERA, continuous erythropoietin receptor activator; Cx, chemotherapy; DP, darbepoetin; EA, epoetin alpha; EB, epoetin beta; ESA, erythropoiesis-stimulating agent; ET, epoetin theta; EZ, epoetin zeta; Fe, iron supplementation; Na, neoadjuvant; Rx, radiotherapy; S, surgery; TF, transfusion; T, transplantation. *Disease control and observations:* AF, adverse effects; CR, complete remission; DFS, disease-free survival; Hb, hemoglobin; HRQoL, health-related quality of life; NR, not reported; NT, neurotoxicity; OS, overall survival; PFS, progression-free survival; PRO, patient-reported outcomes; RFS, relapse-free survival; TE, thrombotic events; TR, tumor response; ↑/ ↓/ ↔, increased/decreased/no significant difference*.

*Studies with indicated negative effects are marked in gray*.

Clinical studies exploring the prognostic significance of EPO and EPOR expression in cancer patients have also been explored and are listed in Table 2. Several studies indicated increased EPO expression as prognostic factor for reduced overall survival (120, 177) or vice versa, no EPO expression as prognostic factor for increased overall survival (178, 179). Furthermore, increased EPOR expression was identified as prognostic factor for reduced overall survival, more aggressive tumor behavior, and progression-free survival (57, 73, 120, 180–182). Increased EPOR expression was also linked to reduced response to anti-cancer treatment (56, 57). While no or reduced EPOR expression was identified as prognostic factor for increased overall survival (178) and in contrast, also to increased cancer recurrence rate (183). Most of these studies did not characterize the subtype of EPOR isoforms, which is crucial for interpretation of the data, as indicated by Küster et al. (183).

**Table 2:** **Clinical studies exploring prognostic significance of EPO and EPOR expression in cancer patients**.

Study (reference)	No. of patients (cancer type)	Therapy	EPO expression as PF (method)	EPOR expression as PF (method)	Other observations
Seibold et al. (178)	*N* = 114 (head and neck SCC)	S, Rx,	IPF – no EPO: ↑LRC, ↑MFS, ↑OS	IPF – no EPOR: ↑OS	
Lin et al. (180)	*N* = 256 (oral SCC)	S	/	IPF – ↑EPOR: ↑ATB, ↓OS, ↓DSS (qPCR, WB, IHC)	
Wang et al. (120)	*N* = 172 (GAC)		IPF – ↓OS; ↑EPO: ↑EPOR, ↑ATB, ↑MD (IHC)	↑EPOR: ↑ATB, ↑MD (IHC)	↑VEGF- ↓EPOR
Welsch et al. (177)	*N* = 104 (PDAC)		IPF, ↑sEPO: ↓OS (qPCR, ELISA, IHC)	(qPCR, IHC)	
Gombos et al. (184)	*N* = 24 (colorectal AC)		↑EPO (IHC, qPCR, WB)	↑EPOR (IHC, qPCR, WB)	↑EPO and EPOR in ischemia/necrosis
Beschorner et al. (185)	*N* = 43 (CPT)		/	↓EPOR (IHC, qPCR, WB)	
Rades et al. (179)	*N* = 62 (NSCLC)	Rx	IPF; no EPO: ↑LRC, ↑OS		↑EPO and ↑EPOR: ↓PF
Volgger et al. (181)	*N* = 107 (breast)	ESA	/	↑EPOR: ↑ER and ↑PR, ↑CRR, ↔OS (IHC, qPCR, WB)	
Xu et al. (186)	*N* = 96 (prostate: PCa, PIN, BPH)		↑EPO (BPH) (IHC)	↑EPOR (PCa, PIN) (IHC)	
Liang et al. (57)	*N* = 55/37 (breast)	TZ, ESA	/	↑EPOR and ↑HER2: ↓TR to TZ, ↓PFS, ↓OS (IHC)	
Mirmoham-medsadegh et al. (187)	*N* = 20 (melanoma)		/	↑EPOR (qPCR, IHC, WB)	
Giatromanolaki et al. (182)	*N* = 72 (endometry)		/	↑EPOR: ↑ATB, ↓PF (IHC)	↑EPOR- ↑HIF1α- ↑VEGF
Larsson et al. (56)	*N* = 500 (breast: ER + , PR +)	TAM	(qPCR, ELISA)	↑EPOR: ↓TR to TAM (qPCR, IHC)	
Li et al. (119)	*N* = 65 (tongue SCC)	S	IPF (IHC)	IPF (IHC)	
Miller et al. (73)	*N* = 159 (various)	ESA	(qPCR)	↔PFS, ↑EPOR: ↓PFS in unresected T (qPCR)	JAK2, HSP70
Küster et al. (183)	*N* = 131 (meningioma)		/	↓EPOR: ↑CRR (IHC, qPCR, WB)	EPOR-F, EPOR-T, EPOR-S

**Disease/cancer type*: BPH, benign prostatic hyperplasia; CPT, choroid plexus tumors (glioma, meningioma); ER+, estrogen receptor positive; GAC, gastric adenocarcinoma; NSCLC, non-small-cell lung cancer; PR+, progesterone receptor positive; SCC, squamous cell carcinoma; PCa, prostate carcinoma; PDAC, pancreatic ductal adenocarcinoma; PIN, prostate intraepithelial neoplasia. *Therapy:* ESA, erythropoiesis-stimulating agent; Rx, radiotherapy; S, surgery; TAM, tamoxifen; TZ, trastuzumab. *Prognostic factor and observations*: ATB, aggressive tumor behavior (TNM stage, T and N classification); CRR, cancer recurrence rate; DSS, disease-specific survival; IPF, independent prognostic factor; LRC, loco-regional control; MD, microvessel density; MFS, metastases-free survival; OS, overall survival; PF, prognostic factor; PFS, progression-free survival; sEPO, serum EPO; TR, tumor response; ↑/ ↓/ ↔, increased/decreased/no significant difference. *Methods*: IHC, immunohistochemistry; qPCR, quantitative PCR; WB, Western blotting*.

Based on discussed clinical studies, the adverse effect of EPO/ESA in cancer patients during anti-cancer therapy could be related to chronic EPO treatment. Such EPO/ESA treatments are long termed and rarely single dosed. We suggest that the concurrent use of long-termed EPO/ESA and anti-cancer treatment is the main reason for EPO/ESA negative effects on response to anti-cancer therapy, overall survival, and disease recurrence. The mechanisms are still not well understood and more profound molecular and biochemical characterization is needed; however, they may be linked to one of the EPO/ESA mechanisms indicated in the previous chapter. EPOR, βcR, and EPHB4 were previously shown to be expressed in various tumors. We do speculate that one of these receptors and/or possible analogs of these receptors may be involved in the response of cancer cells to EPO/ESA therapy.

## Conclusion

A plethora of scientific evidence demonstrates a growth-promoting, anti-apoptotic action of EPO and other ESAs on non-hematopoietic cells, both normal and malignant, and this is supported by numerous clinical observations showing adverse effects of EPO administration on the clinical management of tumor growth and progression. As anticipated just a few years ago (28), physicians who care for anemic cancer patients have been facing a dilemma, whether to treat the anemic patient with an ESA, thereby potentially increasing the risk of worsening the malignancy, or to withhold ESA treatment, with resultant patient fatigue, reduced physical activity, increased hypoxic stress, and reliance on transfusion therapy. Primary tumors are not yet EPOR typed (like breast cancers are assessed for ER/PR expression) though this idea should be considered. There has been much discussion of EPO use in cancer patients, and several professional and regulatory organizations and authorities have issued various guidances. Perhaps, the following “rule” used by several clinicians interviewed by one of the authors should be considered. If the cancer patient is being treated with curative intent, avoid the use of ESAs. If the treatment plan is more conservative or palliative, consider ESAs for anemia treatment, but proceed with great caution.

## Author Contributions

Nataša Debeljak outlined the work. All authors designed and drafted the work. Arthur J. Sytkowski critically revised the work. All authors approved final version of the work and agreed to be accountable for all aspects of the work.

## Conflict of Interest Statement

The authors declare that the research was conducted in the absence of any commercial or financial relationships that could be construed as a potential conflict of interest.
